# Supplementary material for: Unexpected steric hindrance failure in the gas phase F− + (CH3)3CI SN2 reaction
Source: Nat Commun. 2022 Jul 30;13:4427. doi: 10.1038/s41467-022-32191-6 (PMC9338938; doi:10.1038/s41467-022-32191-6)
Supplement: Supplementary file 1 — Supplementary information [file 41467_2022_32191_MOESM1_ESM.pdf]

Supplementary Information for

Unexpected steric hindrance failure in the gas phase $F^- + (CH_3)_3CI$ S_N2 reaction

Xiaoxiao Lu‡,[1] Chenyao Shang‡,[1] Lulu Li,[1] Rongjun Chen,[1] Bina Fu,[1,*] Xin Xu,[2] and Dong H. Zhang[1,†]

[1]State Key Laboratory of Molecular Reaction Dynamics and Center for Theoretical and Computational Chemistry, Dalian Institute of Chemical Physics, Chinese Academy of Sciences, Zhongshan Road 457, Dalian 116023, China‡

[2]Department of Chemistry, Fudan University, Shanghai 200433, China

*Electronic address: bina@dicp.ac.cn

†Electronic address: zhangdh@dicp.ac.cn

‡X. Lu and C. Shang contributed equally to this work.

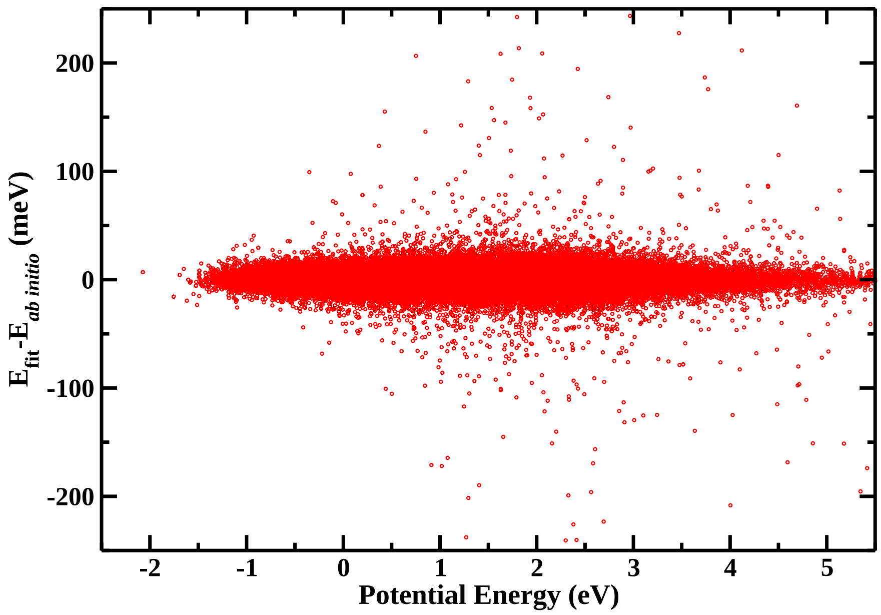

Supplementary Figure 1: Fitting errors for data points on the FI-NN PES, as a function of their corresponding CAM-XYG3/AVTZ(-PP) energies relative to the energy of F^- + $(CH_3)_3CI$.

Supplementary Methods

(1) Potential energy surface

In this work, we constructed a new accurate full-dimensional PES of the F^- + $(CH_3)_3CI$ gas-phase reaction involving the base-induced elimination (E2) and the bimolecular nucleophilic substitution (S_N2) processes. This PES was developed based on a total of ~220,000 CAM-XYG3/aug-cc-pVTZ(aug-cc-pVTZ-PP for iodine atom)[2, 3] energy points and the fundamental invariant nerual network (FI-NN)[4, 5] fitting approach.

For the title organic reactive system consisting of fifteen atoms with multiple heavy atoms, considerable computation effort has to be involved in the electronic structure calculation. In general, the explicitly correlated coupled-cluster (CCSD(T)-F12) method is chosen to gain the benchmark *ab initio* energies. Due to the huge computational cost of the gold standard coupled-cluster calculations, we employed a newly proposed hybrid function CAM-XYG3[2, 3] by Xu and coworkers, which is the combination of the hybrid qualities of XYG3[3] and the long-range correction with the Coulomb-attenuating method (CAM)[2]. This method archives the similar level of accuracy as compared to CCSD(T)/AVTZ(-PP) but significantly reduces the computational effort. It takes about half an hour of real CPU time for a single point using seven-threads in one computer node, indicating this method is ideal for the F^- + $(CH_3)_3CI$ reaction. Besides, the CAM-XYG3

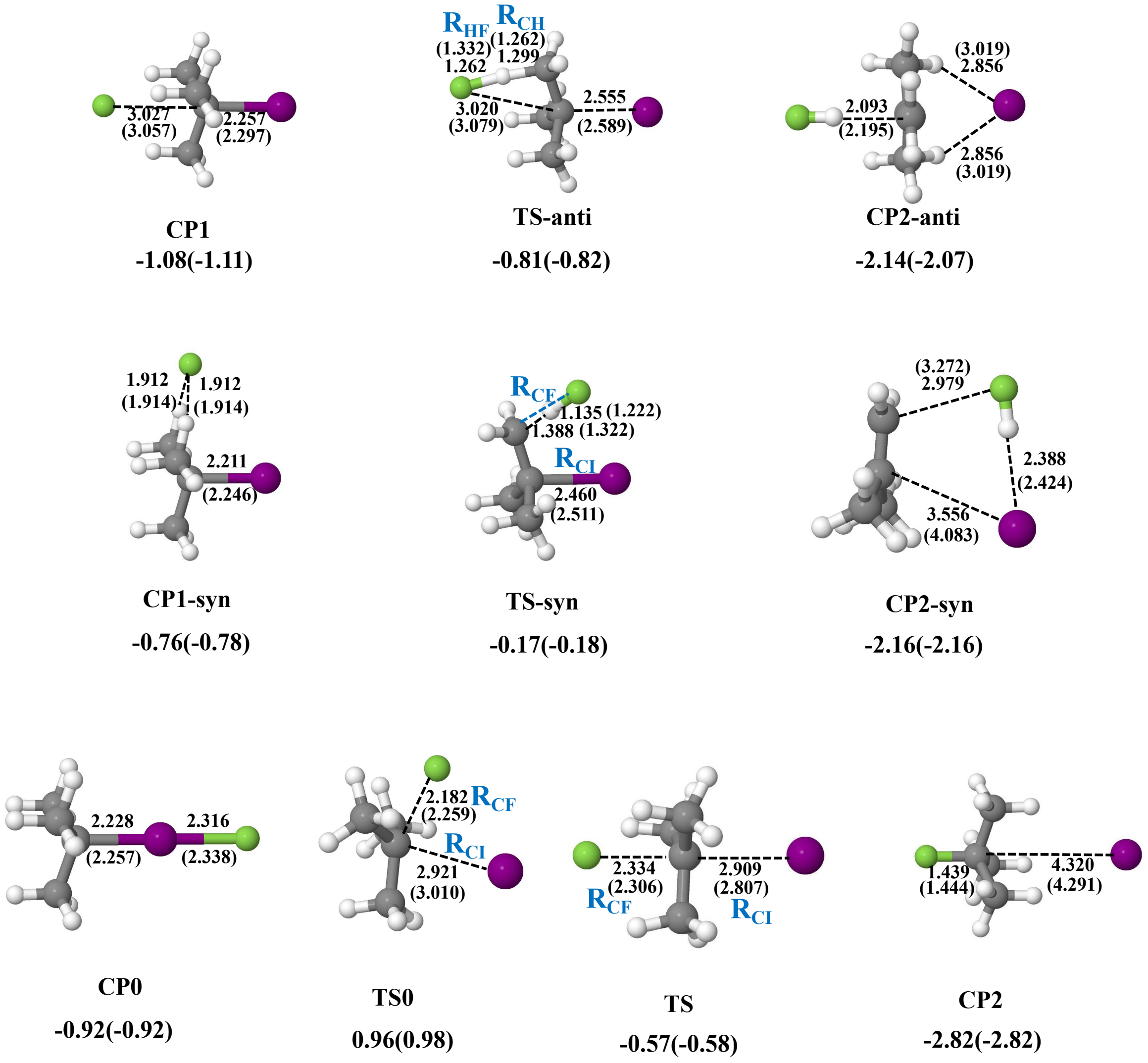

Supplementary Figure 2: Optimized geometries of the stationary points of the E2 and S_N2 reaction paths on the FI-NN PES and from the XYGJ-OS/AVTZ(-PP) (in the brackets) calculations with the key bond lengths (in Å) indicated. The corresponding energies were obtained from the FI-NN PES and the CAM-XYG3/AVTZ(-PP) (in the brackets) method.

representation of long-range interaction between fragments shows good behavior, which plays an important role in the ion-molecular reaction. On the whole, energies of all collected geometries were computed with the hybrid function CAM-XYG3 using Gaussian 09[6], together with Dun-

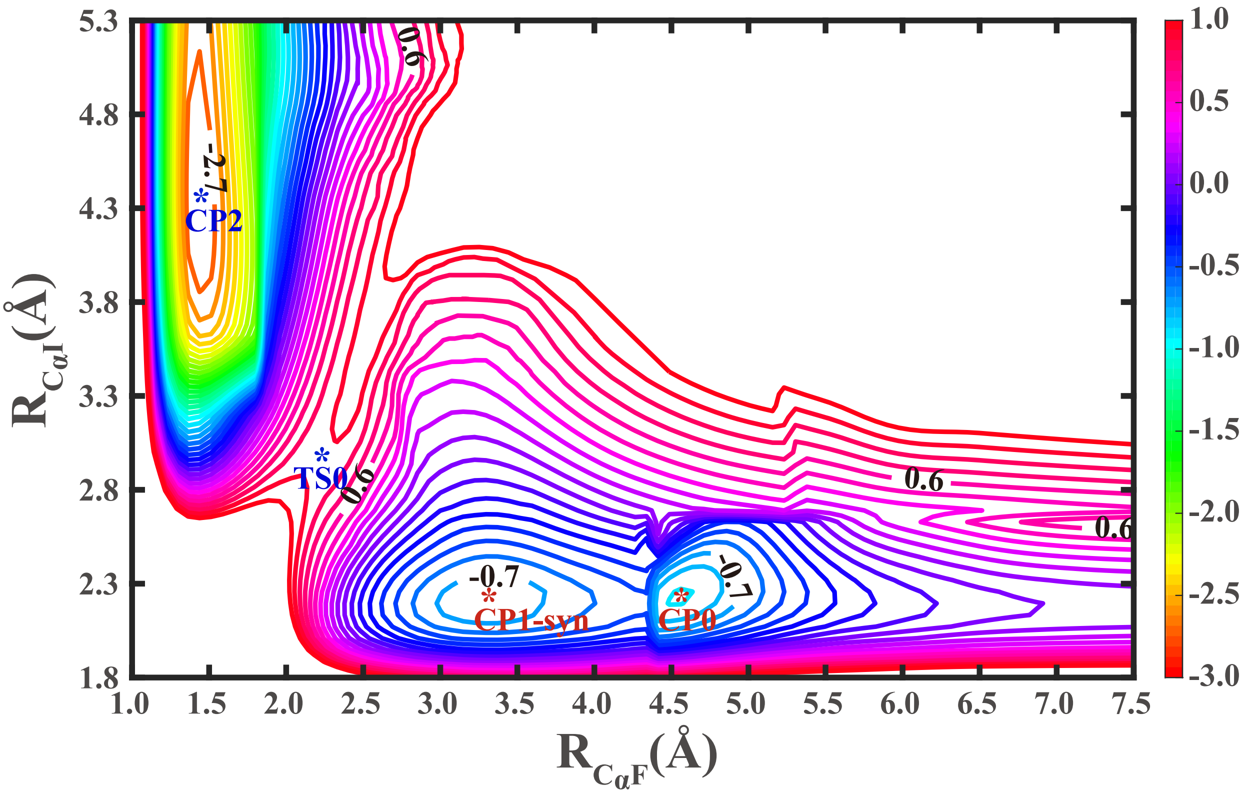

Supplementary Figure 3: The contour plot of front-side attack S_N2 pathway on the FI-NN PES by full-dimensional optimization.

ning's augmented correlation-consistent triple-zeta (aug-cc-pVTZ) basis set and the corresponding aug-cc-pVTZ-PP basis set for iodine atom.

Due to the configuration space of the investigated system is extremely large, we employed the space partitioning and energy splitting methods to overcome the huge difficulties of fitting all the data points. For example, the geometries of the asymptotic region of the reactants F^- + $(CH_3)_3CI$ were collected by the way of energy splitting.

We first constructed an accurate FI-NN tert-butyl iodine ($(CH_3)_3CI$) local PES based on the CAM-XYG3 method. Random configurations of the $(CH_3)_3CI$ reactant were picked from the local PES and combined with the F^- atom. The geometries of the asymptotic region were selected by randomly changing the distance and orientation of the reactants.

We splitted the total energy of a single point into the energy of each reactant and their interaction energy, and then fitted the energies of monomer and interaction energies between the reactants, respectively. The accuracy of the PES with the asymptotic region represented by the

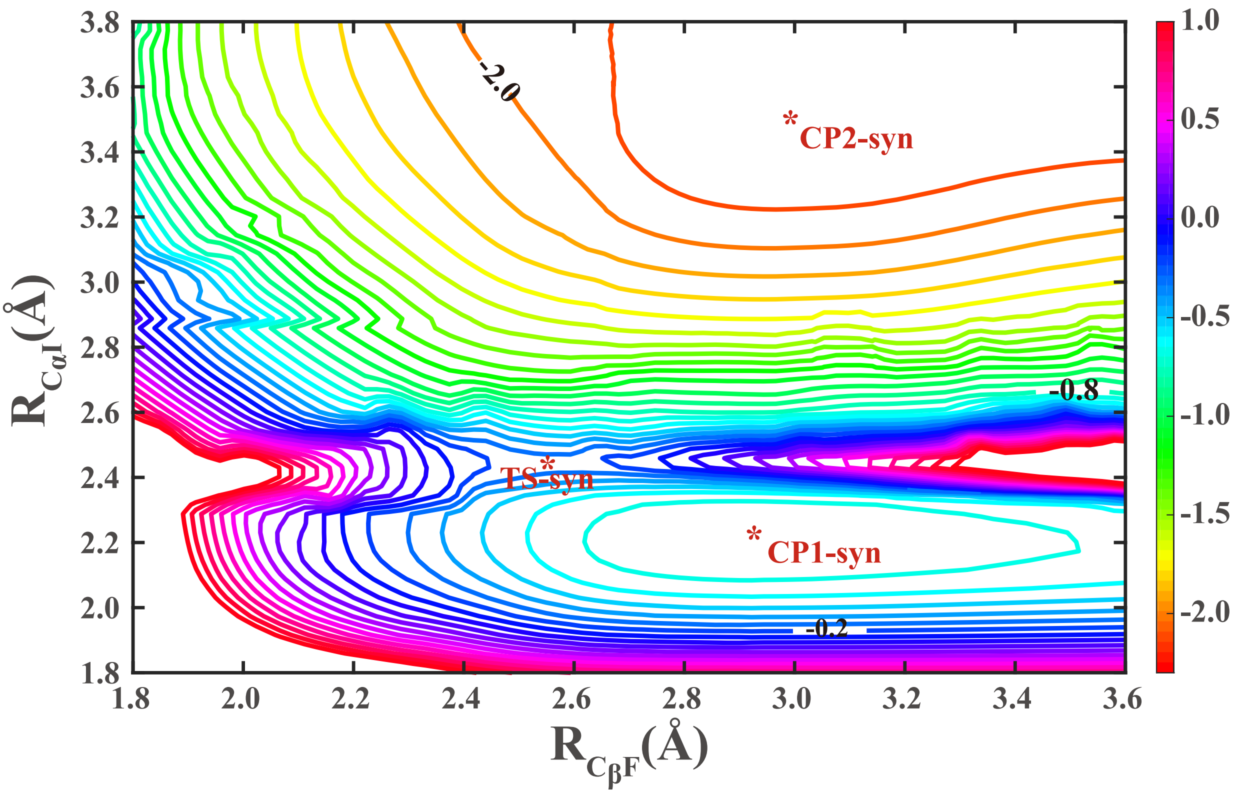

Supplementary Figure 4: The contour plot of syn-E2 pathway on the FI-NN PES by full-dimensional optimization.

combination of the $(CH_3)_3CI$ PES and the interaction energy PES is highly improved. The above strategy is called "energy splitting" method.

Furthermore, we divided the configuration space into three partitions to improve the fitting accuracy and efficiency: 1) the asymptotic region of the reactants (the distance between the center of mass of F^- and $(CH_3)_3CI$ is larger than 7 Å) 2) the interaction region 3) the asymptotic region of the E2 product channel (the minimum distance between the center of mass of three products $(CH_3)_2C{=}CH_2$ + HF + I^-) is larger than 5.5 Å). Adjacent regions was connected smoothly by the switch function. The energies of Part 3 were splitted into the energy of the three fragments and their interaction energies in the same way as the Part 1. The sub-PESs of $(CH_3)_2C{=}CH_2$ and HF were also accurately constructed. With respect to Part 2, we used the direct dynamics simulations based on the unrestricted B3LYP/6-31+G* level of theory to obtained the initial data set starting from the initially guessed transition states of S_N2 and E2. More configurations were iteratively added into the data set by further QCT calculations based the preliminary PES and the updated

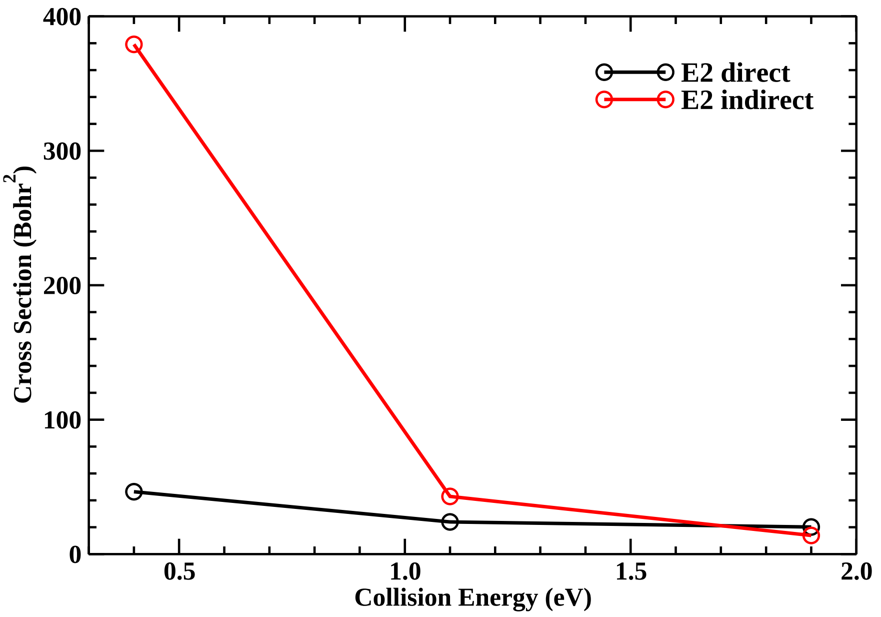

Supplementary Figure 5: Cross sections as a function of the collision energy for the direct and indirect E2 mechanism on the FI-NN PES.

PESs. In addition, the configurations along the minimum energy path and all optimized stationary points were added into the corresponding data set.

Finally, the data points of each part were selected using the following criterion, respectively.

$$D = |E_a - E_b| \sqrt{\frac{1}{n} \sum_{i=1}^{n} (r_{a,i} - r_{b,i})^2}, \quad (1)$$

Here, n represents the number of bond lengths and r_i is the i-th bond length of a specific geometry. The D is an adjustable parameter, here it was set to 0.005. We used the sieve D to discard those data points which are too close in the energy domain and/or geometry domain, in order to improve the efficiency of the FI-NN fitting process. Overall, a total of roughly 50,000, 135,000 and 35,000 data points in the three configuration space parts were calculated using hybrid function CAM-XYG3, respectively.

We used the newly proposed fundamental invariant (FI)-neural network fitting approach [4, 5, 7–12] to guarantee the permutational symmetry of identical atoms. Although FIs can minimize the number of invariants compared to permutationally invariant polynomials (PIP)[13, 14], there are still 1282 FIs with a maximum degree of three without considering the permutation symmetry of H atoms on different methyl groups for the F^-+$(CH_3)_3CI$ reaction. The FIs were truncated at the number of 500, which has proven large enough to get an accurate fit.

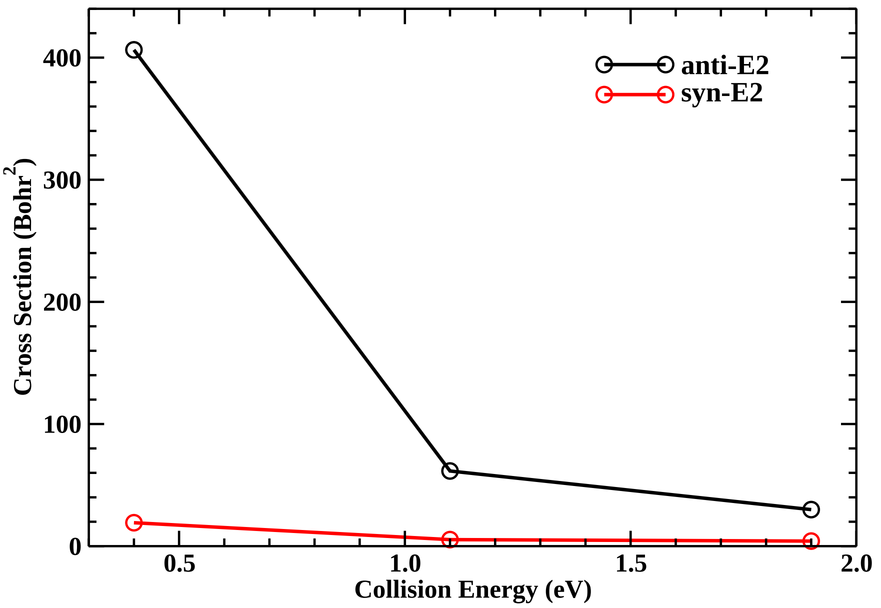

Supplementary Figure 6: Cross sections as a function of the collision energy for the anti-E2 and syn-E2 channel on the FI-NN PES.

Finally, the 500-50-100-1 NN structure was chosen to fit the energy points of the interaction region (Part 2) and the interaction energies between the reactant (Part 1) and product fragments (Part 3). Furthermore, the 746 FIs up to 3 degree were employed to fit the $(CH_3)_3CI$ local PES, together with 50 and 100 neurons on the first and the second hidden layer. Besides, we used the 606-10-100-1 NN structure to train the $(CH_3)_2C{=}CH_2$ PES with all FIs up to degree 3. Note that each bond length x_i in FIs was further replaced by its inverse $1/x_i$, which displays better performance in the fitting accuracy.

In the NN training processes, the Levenberg–Marquardt algorithm[15] was employed to update the weights and biases, which aim to obtain an optimal fit. The root mean square error (RMSE) defined in Equation (2) was applied to measure the fitting error.

$$\text{RMSE} = \sqrt{\frac{1}{n}\sum_{i=1}^{n}(E_{\text{fit}} - E_{ab\ initio})^2} \tag{2}$$

Overall, a total of ~ 135,000 energy points in Part 2 was fitted using the optimal 500-50-100-1 NN structure with 30,251 parameters, resulting in a quite small RMSE of 9.3 meV (0.21 kcal mol^{-1}). In particular, the interaction energies of Part 1 and Part 3 were fitted ending up with an excellent RMSE of 0.9 meV and 3.4 meV, respectively. Two local PESs ($(CH_3)_3CI$ and $(CH_3)_2C{=}CH_2$) are also fitted accurately, whose RMSE is 3.6 meV and 3.8 meV, respectively.

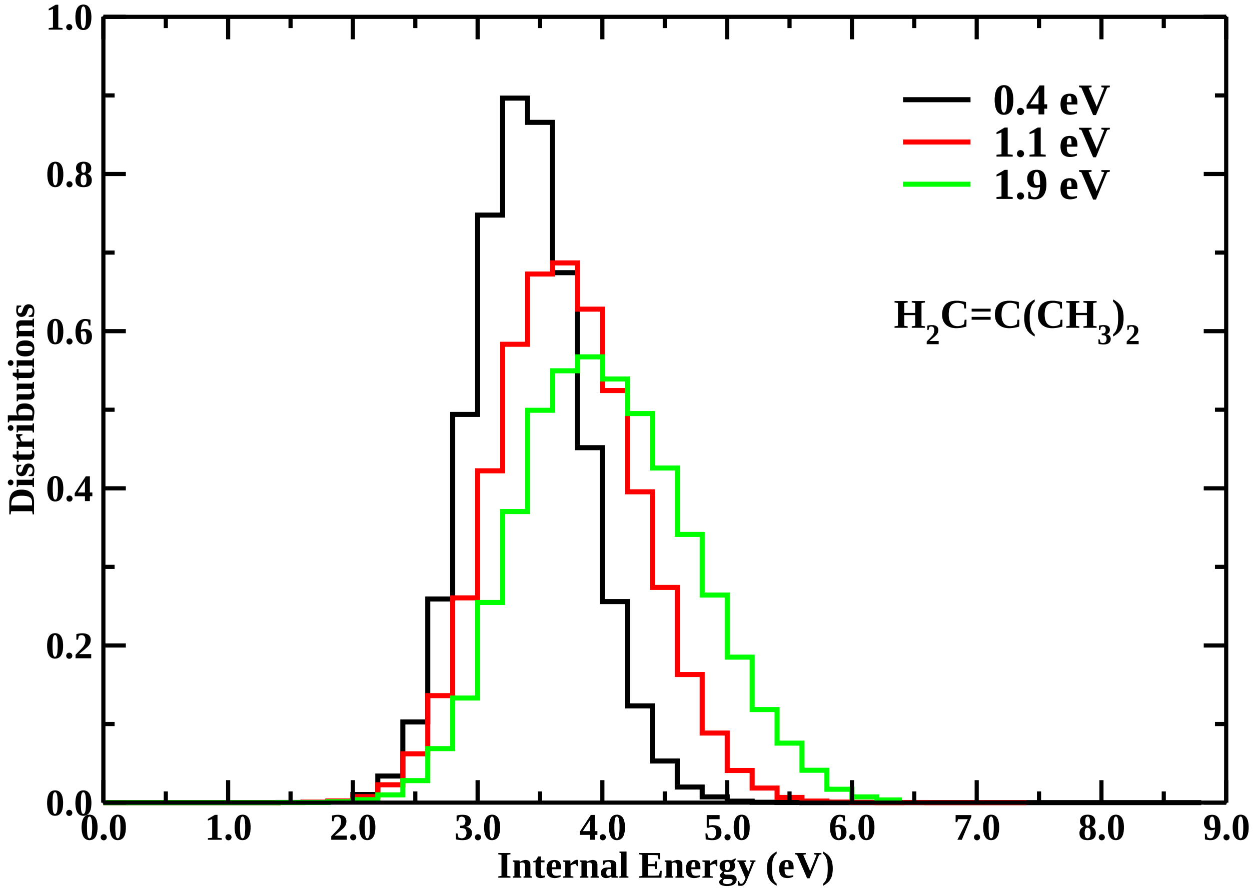

Supplementary Figure 7: The internal energy distributions of product $(CH_3)_2CCH_2$ at differen collision energies.

Overall, the total RMSE of all data points of the three parts is only 8.3 meV. Figure S1 shows the fitting errors of all data points with respect to their corresponding CAM-XYG3/AVTZ(-PP) energies. Although the energy range of the current PES is fairly large (~ 8 eV), the *ab initio* energies are well reproduced by the FI-NN fitting. All the structures of stationary points along the reaction paths are shown in Fig. S2 and the key bond lengths are indicated. It is extremely time consuming to directly make the optimization using CAM-XYG3/AVTZ(-PP), because numerical forces have to be calculated and the analytical forces are not available for this method. Instead, we used the XYGJ-OS[16] method together with the AVDZ(-PP) basis, and found the resulting geometries are in good agreement with those on the PES. The energies obtained from the PES were compared with those calculated by the direct CAM-XYG3/AVTZ(-PP) method, which also shows good agreement.

(2) Quasi-calssical trajectory calculations

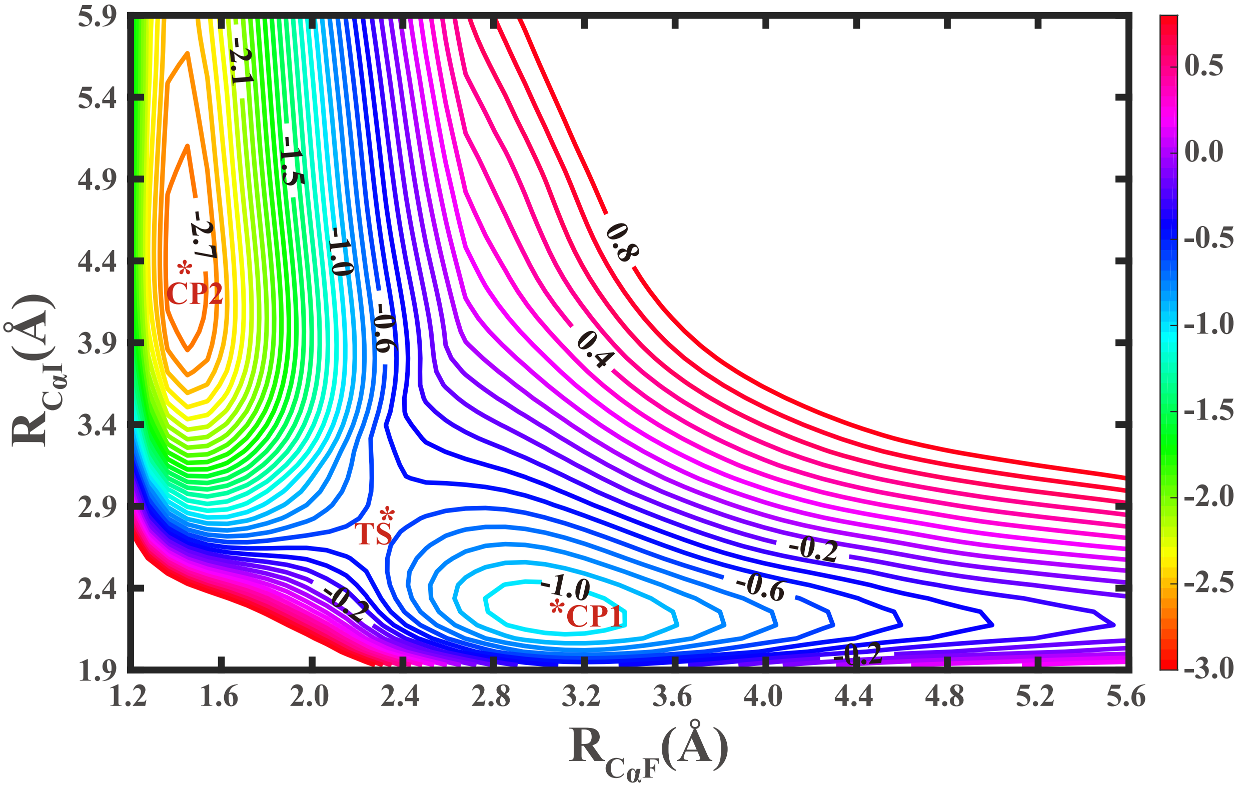

Supplementary Figure 8: The contour plot of back-side attack S_N2 on the modified PES by full-dimensional optimization.

Standard QCT calculations [17, 18] for the F^- + $(CH_3)_3CI$ reaction were carried out at collision energies ranging from 0.2 eV to 1.9 eV on the FI-NN PES, for the $(CH_3)_3CI$ reactant initially in the ground rovibrational state. We randomly sampled the normal coordinates and momenta to get the initial coordinates and momenta of $(CH_3)_3CI$. Adjustments were then made to the momenta to force the angular momentum of $(CH_3)_3CI$ to zero. The initial distance between the center of mass of two reactants was $\sqrt{x^2 + b^2}$, where b is the impact parameter and x was set to 38.0 Bohr due to the large long-range interactions in the entrance channel for such a ion-molecular reaction. Here, the orientation of $(CH_3)_3CI$ was randomly sampled with respect to the F anion. The impact parameter b was scanned from 0 to the maximum impact parameter (b_{max}) with a step size of 0.5 Bohr. The value of b_{max} was determined ranging from 21.0 Bohr to 10.0 Bobr after preliminary tests as the collision energy increases.

Our PES can cover the region where the distance between the centers of the mass of two reactants is 25 Å in the entrance channel. In this work, all trajectories were run using the Velocity-Verlet integration algorithm with a time step of 0.024 fs for a maximum time of 25 ps. We ter-

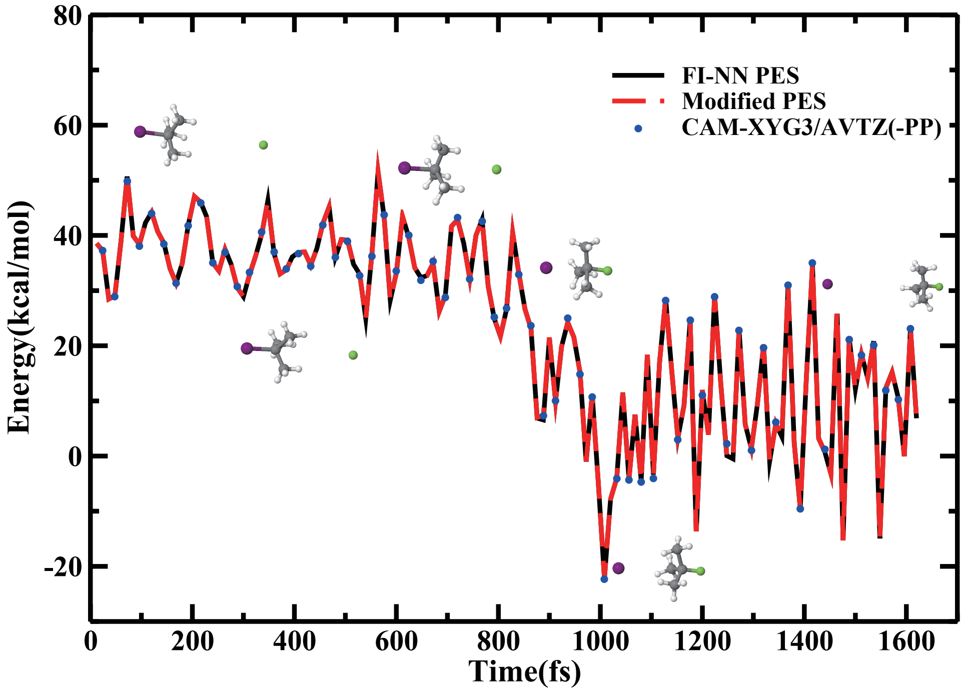

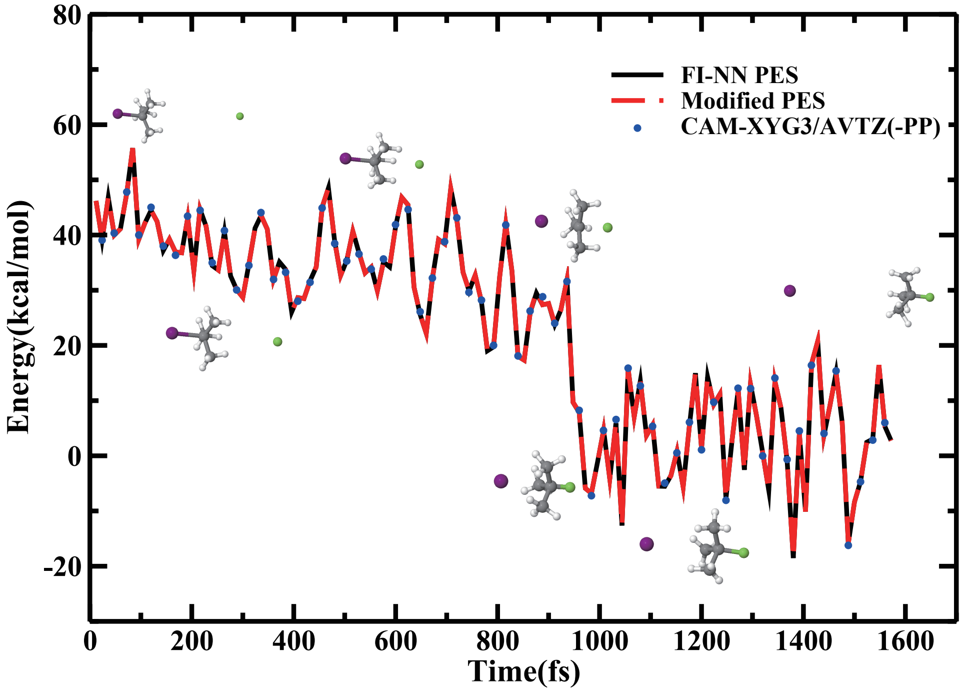

Supplementary Figure 9: The potential energies of configurations along the two randomly selected trajectories.

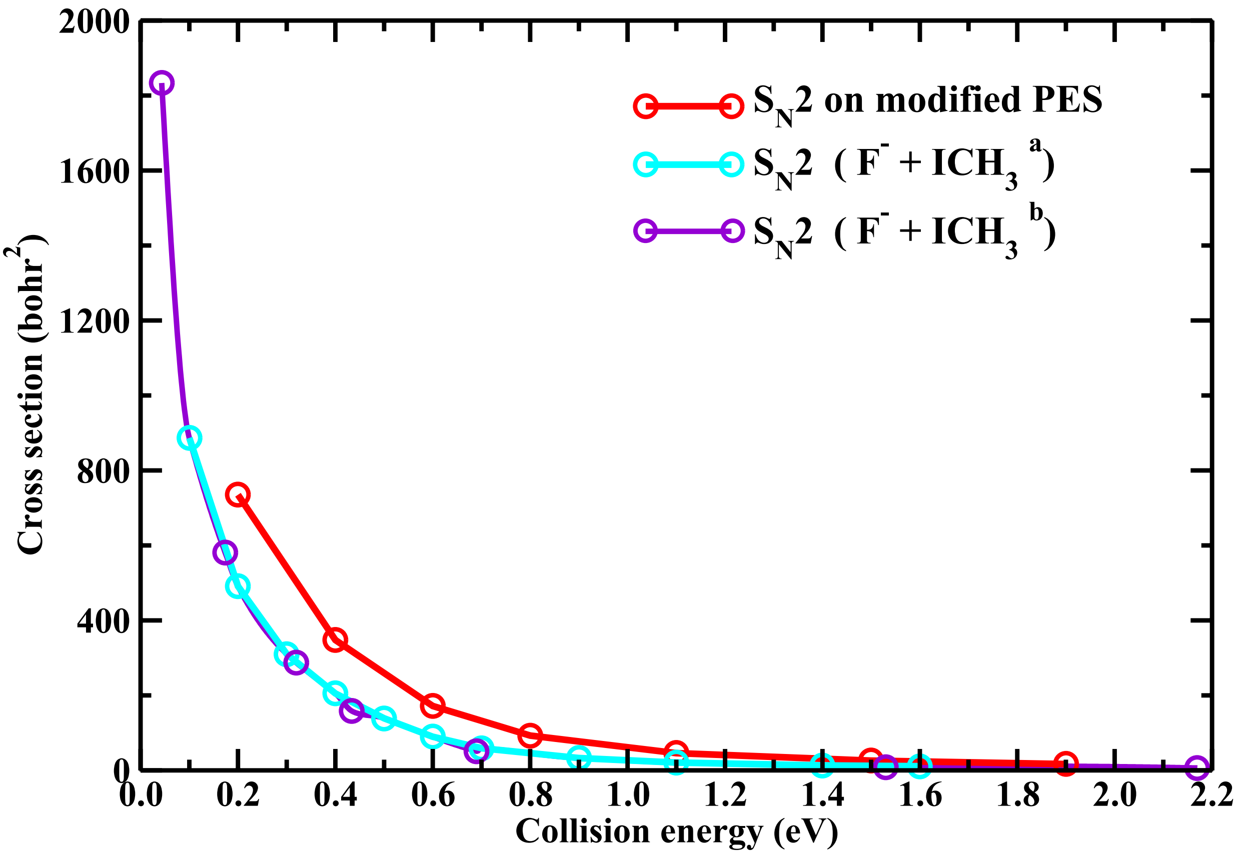

Supplementary Figure 10: Comparisons of the intrinsic S_N2 reactivity of F^- + $(CH_3)_3CI$ on the modified PES and the S_N2 reactivity of F^- + CH_3I on the FI-NN PES[a] as well as on the PIP PES developed by Czakó *et al*[b][1].

minated the trajectory when any two fragments reach a separation of 30 Bohr, ending up with the formation of $(CH_3)_2C{=}CH_2$ + HF + I^- or $(CH_3)_3CF$ + I^- or returning to the reactants. A total of roughly 4.2 million trajectories were run to obtain the detailed dynamical information of the product I^- at each collision energy.

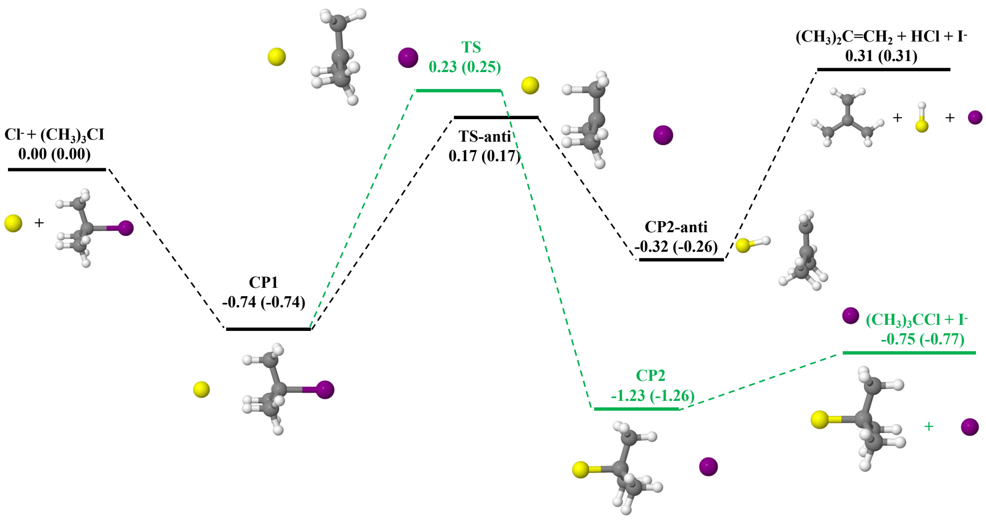

Supplementary Figure 11: PES schematic of the $Cl^- + (CH_3)_3CI$ reaction. The back-side attack S_N2 pathway is indicated by green curve, and the anti-E2 pathway is indicated by black curve.

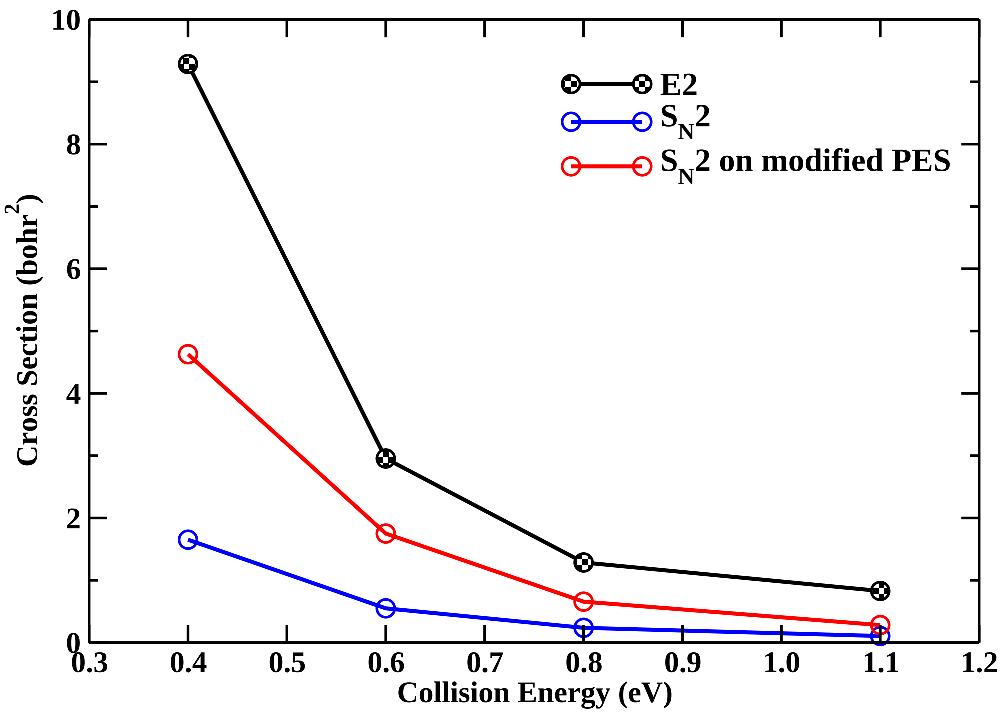

Supplementary Figure 12: Cross sections as a function of collision energy for the E2 and S_N2 reactions for $Cl^- + (CH_3)_3CI$ and those for the S_N2 reaction on the modified PES. A repulsive potential between Cl^- and β-H in the vicinity of the E2 transition state was added in the modified PES, which blocks the E2 pathway but nothing is changed for S_N2 as presented for $F^- + (CH_3)_3CI$.

Supplementary References

[1] Olasz, B., Szabó, I. & Czakó, G. High-level ab initio potential energy surface and dynamics of the F^- + CH_3I S_N2 and proton-transfer reactions. *Chem. Sci.* **8**, 3164–3170 (2017).

[2] Yanai, T., Tew, D. P. & Handy, N. C. A new hybrid exchange–correlation functional using the Coulomb-attenuating method (CAM-B3LYP). *Chem. Phys. Lett.* **393**, 51 – 57 (2004).

[3] Zhang, Y., Xu, X. & Goddard, W. A. Doubly hybrid density functional for accurate descriptions of nonbond interactions, thermochemistry, and thermochemical kinetics. *Proc. Nat. Acad. Sci. USA* **106**, 4963–4968 (2009).

[4] Shao, K., Chen, J., Zhao, Z. & Zhang, D. H. Communication: Fitting potential energy surfaces with fundamental invariant neural network. *J. Chem. Phys.* **145**, 071101 (2016).

[5] Chen, R., Shao, K., Fu, B. & Zhang, D. H. Fitting potential energy surfaces with fundamental invariant neural network. II. Generating fundamental invariants for molecular systems with up to ten atoms. *J. Chem. Phys.* **152**, 204307 (2020).

[6] Frisch, M. J. *et al.* Gaussian~09 Revision E.01 (2009). Gaussian Inc. Wallingford CT 2009.

[7] Lu, X., Shao, K., Fu, B., Wang, X. & Zhang, D. H. An accurate full-dimensional potential energy surface and quasiclassical trajectory dynamics of the H + H_2O_2 two-channel reaction. *Phys. Chem. Chem. Phys.* **20**, 23095–23105 (2018).

[8] Li, L., Fu, B., Yang, X. & Zhang, D. H. A global ab initio potential energy surface and dynamics of the proton-transfer reaction: OH^- + D_2 → HOD + D^-. *Phys. Chem. Chem. Phys.* **22**, 8203–8211 (2020).

[9] Fu, Y.-L. *et al.* Collision-induced and complex-mediated roaming dynamics in the H + C_2H_4 →H_2 + C_2H_3 reaction. *Chem. Sci.* **11**, 2148–2154 (2020).

[10] Lu, X., Fu, B. & Zhang, D. H. Dynamics and kinetics of the OH + HO_2 → H_2O + O_2 ($1\Delta_g$) reaction on a global full-dimensional singlet-state potential energy surface. *Phys. Chem. Chem. Phys.* **22**, 26330–26339 (2020).

[11] Fu, Y.-L., Lu, X., Han, Y.-C., Fu, B. & Zhang, D. H. Supercollisions of fast H-atom with ethylene on an accurate full-dimensional potential energy surface. *J. Chem. Phys.* **154**, 024302 (2021).

[12] Fu, Y.-L., Bai, Y., Han, Y.-C., Fu, B. & Zhang, D. H. Double-Roaming Dynamics in the H + C_2H_2 → H_2 + C_2H Reaction: Acetylene-Facilitated Roaming and Vinylidene-Facilitated Roaming. *J. Phys.*

Chem. Lett. **12**, 4211–4217 (2021).

[13] Braams, B. J. & Bowman, J. M. Permutationally invariant potential energy surfaces in high dimensionality. *Int. Rev. Phys. Chem.* **28**, 577–606 (2009).

[14] Bowman, J. M., Czako, G. & Fu, B. High-dimensional ab initio potential energy surfaces for reaction dynamics calculations. *Phys. Chem. Chem. Phys.* **13**, 8094–8111 (2011).

[15] Hagan, M. T. & Menhaj, M. B. Training feedforward networks with the Marquardt algorithm. *IEEE Transactions on Neural Networks* **5**, 989–993 (1994).

[16] Zhang, I. Y., Xu, X., Jung, Y. & Goddard, W. A. A fast doubly hybrid density functional method close to chemical accuracy using a local opposite spin ansatz. *Proc. Nat. Acad. Sci.* **108**, 19896–19900 (2011).

[17] Hase, W. L. Classical Trajectory Simulations: Initial Conditions. In *Encyclopedia of Computational Chemistry* (ed. Allinger, N. L.), vol. 1, 402–407 (Wiley, New York, 2002).

[18] Hase, W. L. Classical Trajectory Simulations: Final Conditions. In *Encyclopedia of Computational Chemistry* (ed. Allinger, N. L.), vol. 1, 399–402 (Wiley, New York, 2002).
