# Supplementary material for: Unexpected steric hindrance failure in the gas phase F− + (CH3)3CI SN2 reaction
Source: Nat Commun. 2022 Jul 30;13:4427. doi: 10.1038/s41467-022-32191-6 (PMC9338938; doi:10.1038/s41467-022-32191-6)
Supplement: Supplementary file 3 — Description of Additional Supplementary Files [file 41467_2022_32191_MOESM3_ESM.docx]

Description of Additional Supplementary Files

File Name: Supplementary Movie 1

Description: indirect trajectory via anti-E2

File Name: Supplementary Movie 2

Description: direct trajectory via anti-E2

File Name: Supplementary Movie 3:

Description: indirect trajectory via syn-E2

File Name: Supplementary Movie 4

Description: direct trajectory via syn-E2

File Name: Supplementary Movie 5

Description: indirect trajectory via SN2

File Name: Supplementary Movie 6

Description: direct trajectory via SN2
